# Investigation of cold-resistance mechanisms in cryophylactic yeast *Metschnikowia pulcherrima* based on comparative transcriptome analysis

**DOI:** 10.3389/fmicb.2024.1476087

**Published:** 2024-09-25

**Authors:** Zaizhu Yuan, Zhengkai Ge, Qingquan Fu, Fangfang Wang, Qingling Wang, Xuewei Shi, Bin Wang

**Affiliations:** ^1^Key Laboratory of Agricultural Product Processing and Quality Control of Specialty (Co-Construction by Ministry and Province), School of Food Science and Technology, Shihezi University, Xinjiang, Shihezi, China; ^2^Key Laboratory for Food Nutrition and Safety Control of Xinjiang Production and Construction Corps, School of Food Science and Technology, Shihezi University, Xinjiang, Shihezi, China; ^3^Engineering Research Center of Storage and Processing of Xinjiang Characteristic Fruits and Vegetables, Ministry of Education, School of Food Science and Technology, Shihezi University, Xinjiang, Shihezi, China

**Keywords:** cryophylactic yeast, comparative transcriptome, cold-resist mechanisms, RNA-Seq, differential expressed gene

## Abstract

**Introduction:**

Low temperature inhibits the growth of most microorganisms. However, some microbes can grow well in a low temperature, even a freezing temperature.

**Methods:**

In this study, the mechanisms conferring cold resistance in the cryophylactic yeast *Metschnikowia* (*M.*) *pulcherrima* MS612, an isolate of the epidermis of ice grapes, were investigated based on comparative transcriptome analysis.

**Results:**

A total of 6018 genes and 374 differentially expressed genes (> 2-fold, *p* < 0.05) were identified using RNA-Seq. The differentially expressed genes were mainly involved in carbohydrate and energy metabolism, transport mechanisms, antifreeze protection, lipid synthesis, and signal transduction. *M. pulcherrima* MS612 maintained normal growth at low temperature (5°C) by enhancing energy metabolism, sterol synthesis, metal ion homeostasis, amino acid and MDR transport, while increased synthesis of glycerol and proline transport to improve its resistance to the freezing temperature (−5°C). Furthermore, cAMP-PKA and ERAD signaling pathways contribute to resist the low temperature and the freezing temperature, respectively.

**Conclusion:**

This study provides new insights into cold resistance in cryophylactic microorganisms for maneuvering various metabolism to resist different cold environment.

## 1 Introduction

More than 80% of the earth’s environments exhibit temperatures below 5°C. This is particularly true for deep seas, ice caps, permafrost regions, and alpine glaciers (Margesin and Collins, 2019). Low temperatures and environmental temperature changes are common stressors experienced by all organisms, from microbes and fungi to plants and animals. The response of prokaryotes and eukaryotes to cold stress has been widely investigated in a large number of organisms and cellular models (Al-Fageeh and Smales, 2006). Low temperature limits microbial growth by changing the physical state of water and decreasing the activity of key cellular enzymes. Further, the oxidative stress caused by the increase in free radicals destroys the activity of biological macromolecules such as proteins, nucleic acids, and lipids, resulting in the biological loss of cell function (García-Ríos et al., 2016). Furthermore, low-temperature conditions are often accompanied by other stressors, such as high osmotic pressure, high levels of UV radiation, and nutrient scarcities, making the extreme habitats very unfit for survival (Buzzini and Margesin, 2014). However, low-temperature environments offer an opportunity for physiological adaptation, enabling cold-tolerant microorganisms to combat low-temperature stress and survive better at extreme temperatures (Siddiqui et al., 2013; Maayer et al., 2014; Boetius et al., 2015).

Yeasts, one of the most common microorganisms found in nature, are widely present in various harsh niches and generally show a strong tolerance to low temperatures (Boetius et al., 2015). *Saccharomyces* (*S.*) *cerevisiae* contributing to alcohol fermentation has been used as a classical model to investigate cold resistance mechanisms (García-Ríos et al., 2017). When exposed to a low temperature, *S. cerevisiae* maintains cell membrane fluidity by changing the composition of membrane phospholipids (Rodríguez-Vargas et al., 2007), increases membrane and protein stability through accumulating a high level of trehalose (Benoit et al., 2009), decreases the intracellular and extracellular osmotic pressure imbalance by enhancing glycerol synthesis (Panadero et al., 2006), prevents protein denaturation and misfolding through synthesizing molecular chaperones (Panadero et al., 2006), and induces cold shock proteins (Al-Fageeh and Smales, 2006) and antifreeze proteins (Wong et al., 2019). Besides the mechanisms of cold resistance in *S. cerevisiae*, membrane sterol metabolism, activation of the endoplasmic reticulum (ER)-related degradation pathway (ERAD) (Loertscher et al., 2006), and production of cold-active enzymes also contribute to cold resistance in organisms (Daskaya-Dikmen et al., 2018).

In addition to low temperatures, repeat freeze–thaw cycles also provide an ecological challenge to microorganisms (Cabrera et al., 2020). *Metschnikowia* (*M.*) *pulcherrima* strains are dominant consortium on post-harvest fruits and vegetables, because they restrict the growth of other microorganisms through iron competition and can survive repeat freeze–thaw cycles (Wang S. et al., 2020). Interestingly, *M. pulcherrima* possesses potential wide-scale applications in winemaking, because it can reduce the alcohol, acetic acid, and hydrogen sulfide content of wines (Contreras et al., 2015) and increase the fullness and complexity of their taste by synthesizing more terpenes, esters, and fatty acids (Martin et al., 2010; Li et al., 2018). Furthermore, it shows extensive and effective antifungal activity against wild spoilage yeasts such as *Hanseniaspora*, *Pichia*, and *Brettanomyces*, which are undesirable during the winemaking process (Oro et al., 2014).

In this study, *M. pulcherrima* MS612 (MS612) was isolated from the skin of ice grapes in Ili, Xinjiang, where local temperatures range from −15°C to 10°C during the harvest period. Though the potential applications of *M. pulcherrima* in low temperature fermentation have gradually been uncovered (Santamauro et al., 2014; Li et al., 2018), it remains unclear how this yeast survives at a low temperature. To investigate the mechanisms underlying cold resistance in *M. pulcherrima*, the transcriptional expression profile of *M. pulcherrima* under low temperatures was analyzed by using RNA-Seq, obtaining theoretical understanding of cold-tolerance mechanisms in cryophylactic yeast.

## 2 Materials and methods

### 2.1 Strains

*M. pulcherrima* MS612 (MS612) was isolated from the skin of ice Vidal grapes grown in the vineyard of the Yizhu wine factory, Ili, Xinjiang, China [E81.26 N43.49] and stored at the Food Biotechnology Laboratory of Shihezi University (some information on MS612 provided in Supplementary Figures S1, S2). Ice grapes are harvested from December to January, most microorganisms isolated from the skin of ice grapes can grow below 5°C, but the growth rate of MS612 is significantly higher than that of other isolated strains (some details of microbial isolation are shown in Supplementary Figure S1D). Isolation and identification of MS612 was according to a previous literature (Wang et al., 2022). Briefly, the samples were weighed (20.0 g) and added to sterile water (200 mL) for culture (10°C at 200 rpm for 2 h) in an incubator (Boxun Co. Ltd., Shanghai, China). The culture medium (diluted to 10^−1^, 10^−2^, 10^−3^, 10^−4^, 10^−5^, respectively) was evenly coated on a YPD medium plate, and yeast colonies were identified as *Saccharomyces cerevisiae* by microscope (Zhengxi Instrument Equipment Co. Ltd., Shanghai, China) were separated and cultured for 2–3 generations to obtain pure yeast.

### 2.2 Effect of different initial temperatures on the growth conditions of MS612

To investigate the mechanisms conferring cold resistance to MS612, the cells were cultured at seven different temperatures (−5°C, 0°C, 5°C, 10°C, 15°C, 20°C, and 25°C) in Yeast Peptone Dextrose (YPD) medium (AOBOXA, China), and the growth was monitored. Cell growth was examined using spectrophotometry based on the method described by García-Ríos et al. (2016). Overall yeast growth was calculated as the maximum specific growth rate (μ_max_) and the area under the optical density (OD) vs. time curve (AUC) (Zwietering et al., 1990; Aguilera et al., 2007). All the experiments were conducted in triplicate. Meanwhile, the special *S. cerevisiae* EnartisFerm Top15 strain (EnartisFerm, Italia) was used as the growth control as it is suitable for commercial ice winemaking. Three temperatures with obvious growth differences were selected to compare the growth of the two yeasts at different temperatures.

### 2.3 Sample preparation

The lyophilized strains were inoculated into 250 mL conical flasks containing 150 mL YPD medium (2% glucose, 2% peptone, and 1% yeast extract), and cultured at 20°C (with shaking at 150 rpm) until they reached the logarithmic growth phase. Then, 10 mL of activated cell solution was inoculated into 150 mL of fresh YPD medium and cultured at 20°C (shaking at 200 rpm) until the mid-log phase (OD = 1.2–1.5), and the seed solution was prepared. Logarithmic metaphase cultures (10^6^ cells) were inoculated into nine 250 mL conical flasks containing 150 mL of fresh YPD and placed at 20°C, 5°C (cold stress), and −5°C (freezing stress) for durations of 6 h (early stage) and 24 h (late stage) each (Wong et al., 2019; Supplementary Figure S3). Yeast cells were centrifuged at 3,000 rpm at 4°C for 10 min, and then the enriched yeast cells were placed in liquid nitrogen for 1 min and quickly stored at −80°C (Wong et al., 2019). The expression levels under cold treatment were normalized using those observed at 20°C to calculate the fold-change value.

### 2.4 RNA extraction, cDNA library construction, and RNA sequencing

Total RNA was extracted using TRIzol reagent (Invitrogen, Carlsbad, CA, USA) and was treated with the RNase-free DNase set (Qiagen) to remove genomic DNA according to the manufacturer’s instructions (Zhang et al., 2016). The concentration and purity of total RNA were checked using Nanodrop 2000 (Thermo, USA), and the RNA Integrity Number (RIN) values of all samples were measured using Agilent 2100 (Agilent, USA). High-quality RNA samples (OD260/280 ≥ 1.8, OD260/230 ≥ 2.0, RIN > 6.5, 28S:18S > 1.0, >10 μg) were reserved and used for the construction of the sequencing library. The cDNA libraries were sequenced on an Illumina NovaSeq 6000 platform (Illumina Inc., USA) by Biomarker Technology Co., Ltd. (Majorbio, Shanghai, China).

### 2.5 Transcriptomic analysis

Raw data from the Illumina sequencing platform were trimmed using SeqPrep and Sickle. Clean data were obtained by removing sequences containing adapter-dimer reads, low-quality reads (<20 nucleotides), reads with an N ratio (the number of unknown nucleotides/the number of total nucleotides) > 5%, and reads containing more than 20% of low-quality nucleotides (Phred quality score < 10) (Cheng et al., 2014; Zhang et al., 2016). Using TopHat v2.0.9, Almost 78.86–82.07% of the clean reads in each sample could be mapped to the Metschnikowia pulcherrima ASM421770v1 genome (GCA_004217705.1) indicating that the selected genome is reasonable. Differential expression analysis was performed using the DESeq2 tool. Reads Per Kilo bases per Million reads (RPKM) was employed to calculate gene expression levels, and RPKM of three replicates was averaged (Liu et al., 2021). The values in the matrix input were un-normalized counts of sequencing fragments. Default parameters *p* < 0.05 and |log_2_FC| ≥ 1 were set as the threshold indicators for significant differential expression unless specified (Wong et al., 2019; Liu et al., 2021; Heo et al., 2022).

### 2.6 Gene ontology and enrichment analysis

Gene ontology (GO) analysis was performed for each differentially expressed genes (DEGs) using the Blast2 Go software (version 2.3.4) with the default parameters. Blast2 Go was also used for GO functional enrichment analysis of certain genes through Fisher’s exact tests with robust false discovery rate correction to obtain an adjusted suitable *p*-value (for the relationship between certain test gene groups and the whole annotation) (Wong et al., 2019). Correlation and hierarchical clustering analysis were carried out via the AMAP library in R x64 4.1.0. Functional enrichment analysis for GO and KEGG pathway analysis was conducted using Gene Ontology Database and DAVID^2^, respectively (Zhang et al., 2016; Wong et al., 2019).

### 2.7 RT-qPCR analyses

To confirm the transcriptional levels of several genes determined by transcriptome analysis at different temperatures for different durations, reverse transcription coupled to the quantitative polymerase chain reaction (RT-qPCR) was performed using MX3000p (Stratagan, California, USA) using a SuperReal PreMix Plus (SYBR Green) Kit (Tiangen, Beijing, China). All tests were conducted in triplicate. Total RNA extraction, DNase treatment, and cDNA synthesis were performed as described above. Thermocycling was conducted as described previously (Arismendi et al., 2015; Zhang et al., 2016). Transcript levels were normalized using CP034458.1 as reference gene. The primers used in these experiments are listed in Supplementary Table S1. Relative gene expression levels were calculated using the 2^−ΔΔCT^ method (Zhang et al., 2016).

### 2.8 Metabolite measurements

#### 2.8.1 Glycerol measurements

To corroborate the transcriptomic results, *M. pulcherrima*s’ glycerol content was assessed. Intracellular glycerol was determined with reference to the literature (Baltanás et al., 2013). Briefly, yeast cells were incubated in SC medium supplemented with 1 M sorbitol (SCS) at 30°C overnight. Starting from the diluted culture solution, the next day an A600 nm of 0.2 was obtained. The cultures were filtered and resuspended in fresh medium. Next, we split the culture into three flasks. After 300 min, we filtered 5 mL of cells (HAWP02500, Millipore filtration) and collected the flow-through cells in 15 mL falcon tubes. Finally, glycerol concentrations were measured using high-Ph anion-exchange chromatography and pulsed amperometric detection in an ICS-3000 chromatography system (Dionex), as described previously. we collected samples and added cycloheximide to a final concentration of 100 μg/mL; measured absorbance; and imaged the cells to quantify cell volume and reporter gene expression.

The following mathematical transformation was used to calculate the amount of glycerol produced per cell:


$$\begin{array}{l} \frac{\mathrm{Glycerol}\left(\mathrm{pmols}\right)}{\mathrm{cell}}|tx=\frac{{\left[\mathrm{Glycerol}\right]}_{tx}\left(\mathrm{ng}/\mathrm{\mu L}\right)}{ODtx}\\ \times \left(\frac{OD=1}{3\times 10^{7}\mathrm{cells}/\mathrm{mL}}\right)|\mathrm{Vrel}=1\times {\mathrm{Vrel}}_{tx}\times \left(\frac{10^{6}\;\mathrm{\mu L}}{1\;\mathrm{mL}}\right) \end{array}$$

where Vrel_tx_ is the relative volume of cells at a certain time (tx) relative to the volume of cells at time zero.

#### 2.8.2 Proline measurements

Intracellular proline was determined using the Proline Content Assay Kit BC0295 (Solarbio, Beijing, China), as previously reported (Dunayevich et al., 2018). Except for the transfer buffer: we used tri-glycine, methanol, as described for conventional PAGE.

#### 2.8.3 Measurement of iron content

The iron content in the medium was determined using phenanthroline colorimetry (Hsu et al., 2011). Briefly, cells were collected by centrifugation, washed with ddH_2_O, and resuspended with 500 μL of 3% nitric acid. The cell suspension was boiled for 2 h to allow complete digestion of the cells, and cell debris was removed by centrifugation. Iron-containing supernatants (400 μL each) were collected and mixed with 38 mg/mL sodium ascorbate (Sigma) 160 μL, 1.7 mg/mL BPS 320 μL, and 4 M ammonium acetate 126 μL. The chelation reaction mixture was incubated for 5 min at room temperature. The OD_535_ of the BPS-Fe complex was recorded using a spectrophotometer against a blank containing all reagents except cells. To eliminate non-specific absorbance, the OD was subtracted from the OD_535_. The value of iron content was adjusted normalized to the number of digested cells. The number of cells was expressed as OD_600_ with the following formula: (OD_535_ − OD_680_)/(OD_600_). And a standard curve (*y* = 0.0357*X* − 0.0329, *R*^2^ = 0.9972) was created using a FeSO_4_ standard solution.

### 2.9 Statistical analyses

Sample averages were compared using the Student’s t-test and ANOVA, and all tests were performed using SPSS 20.0 (SPSS Inc., Chicago, IL, USA). TBtools was used to generate a heatmap to analyze the trend in gene expression during cold stress (Chen et al., 2022). Histogram and line charts were drawn using Origin 2022 (OriginLab, USA).

## 3 Results and discussion

### 3.1 Effect of cold stress on MS612 growth

As described previously, low temperature can inhibit the growth of most microorganisms (Buzzini and Margesin, 2014). To investigate the effect of temperature on the growth of MS612, it was cultured under seven different temperatures (−5°C, 0°C, 5°C, 10°C, 15°C, 20°C, and 25°C). The suitable temperature range for MS612 growth was found to be 15–25°C (Supplementary Figure S4). In contrast, at 5°C and 0°C, the lag phase of MS612 was prolonged to 24 h. At −5°C, the lag period of MS612 was extended to 72 h, which was harmful to the growth of the strain. To accurately assess the effect of temperature on the growth of MS612, growth parameters (μ_max_ and AUC) were calculated (Supplementary Table S2). The highest maximum specific growth rate was observed at 20°C, but the AUC at 20°C was slightly lower than that at 25°C due to the postponement of the retardation period. At 5°C, the growth rate of MS612 was significantly inhibited. Although the maximum specific growth rate of MS612 was lower than that of the *S. cerevisiae* EnartisFerm Top15 (Top15) strain, its AUC was higher, indicating that some special growth mechanisms may be made MS612 enter the exponential phase faster (Supplementary Figure S5; Supplementary Table S2). At −5°C, MS612 showed growth after 3 days of the adaptive phase and reached its peak growth after 8 days. In contrast, the growth of Top15 almost stopped under these conditions. The rate of cell growth inhibition increased gradually but significantly from 20°C to 5°C and further to −5°C (Supplementary Figure S4). To better understand the potential cold-adaptation mechanisms of MS612, we used comparative RNA-Seq-based transcriptome analysis of yeast grown at 20°C, 5°C and −5°C.

### 3.2 Transcriptome sequencing and assembly

A total of 18 cDNA libraries were prepared from the MS612 after exposure to 20°C, 5°C and −5°C for 6 h and 24 h with three replicates and subjected to Illumina deep sequencing. By comparative transcriptome analysis, a total of 846.4 million raw reads were generated by Illumina paired-end sequencing. After cleaning and quality checks, 841.4 million clean reads were obtained, with an average of 46.7 million reads per sample. All Q30 percentages for the sequences (with an error probability of 0.01; a high-quality indicator) in the 18 libraries were over 94%. The raw data generated from the 18 libraries were shown in Supplementary Table S3 and was deposited in the NCBI-SRA database (PRJNA682923).

### 3.3 DEGs analysis

**Figure 1:**
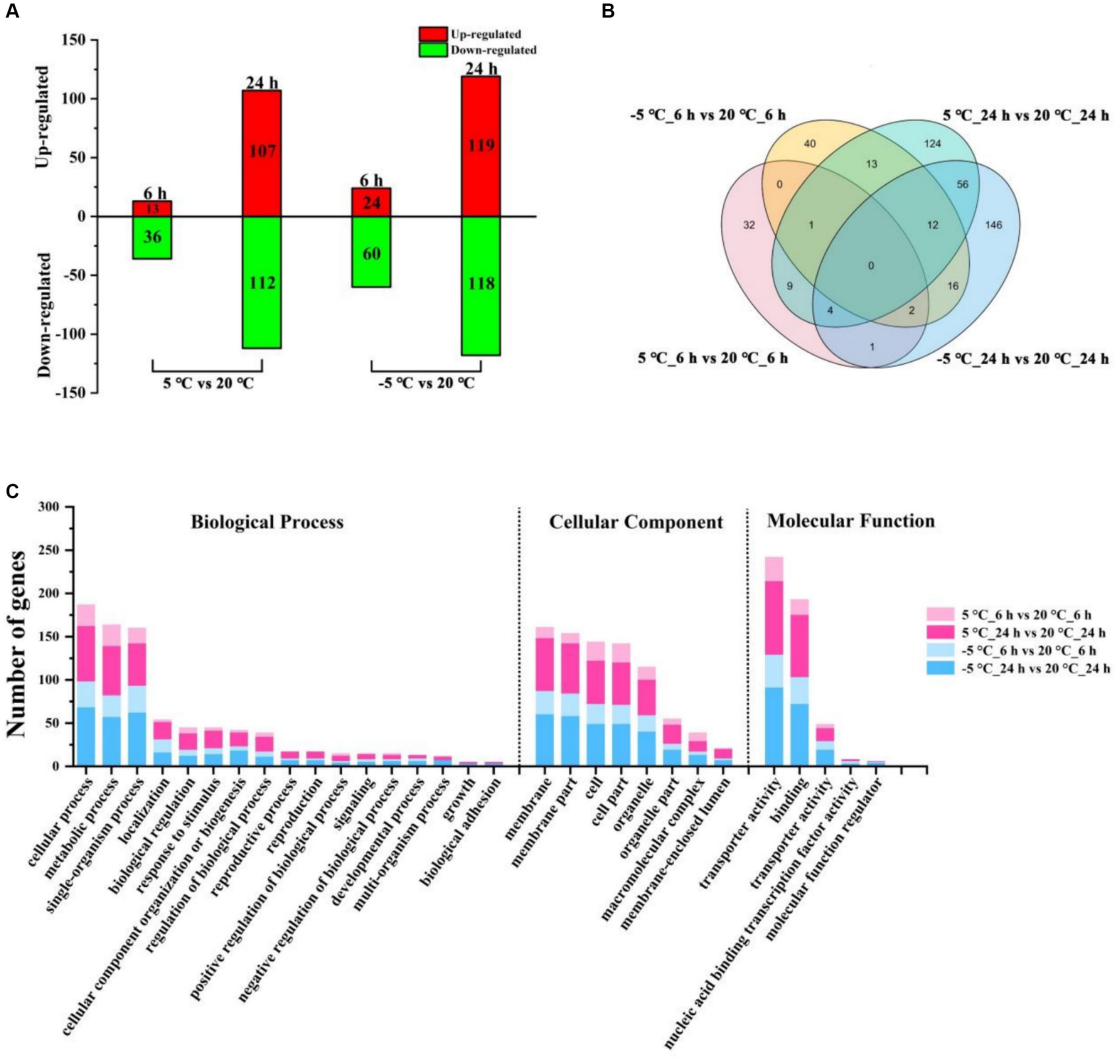
Distribution and functional classification of DEGs under different conditions. **(A)** The number of DEGs of MS612 under different cold treatment after 6 h and 24 h of cold treatment. **(B)** Venn diagram of identified DEGs for MS612 under different cold treatment at different time points. **(C)** GO annotation of DEGs in MS612 under different cold treatment at different time points.

To identify differentially expressed genes (DEGs), we compared transcript levels in MS612 grown at 20°C, 5°C, and −5°C before and after cold stress to identify DEGs. Through comparative transcriptome analysis, a total of 6,018 genes were detected, including 5,784 known and 234 unknown-function genes; 7,684 transcripts were expressed, including 5,738 known and 280 unknown-function transcripts (Supplementary Tables S4, S5). We used |log_2_FC| ≥ 1 and *p* < 0.05 as the cut-off points for identifying up-regulated and down-regulated genes and found a total of 268 DEGs (6 h: 49 [13 up-regulated and 36 down-regulated] and 24 h: 219 [107 up-regulated and 112 down-regulated]) in 5°C and 321 DEGs (6 h: 84 [24 up-regulated and 60 down-regulated] and 24 h: 237 [119 up-regulated and 118 down-regulated]) in −5°C after cold treatment (Figure 1A; Supplementary Table S6).

The data showed that the number of DEGs detected at 24 h after cold treatment was greater than detected at 6 h, indicating that more genes were differentially expressed to cope with low-temperature stress during the cold-adaptation phase and that a long time was needed for these DEGs to get activated and expressed. The few DEGs observed during the cold shock stage may be key genes required to modulate the DEGs observed during the cold-adaptation stage. Interestingly, as the temperature decreased, the number of DEGs increased: the number of DEGs in the cold shock phase at −5°C was twice that observed at 5°C, although the number of DEGs during the cold-adaptation stage was similar at different temperatures. These results suggest that it is crucial for cells to survive under different degrees of cold stress and to respond accordingly during cold shock. At the same time, even if no obvious cells growth was observed at −5°C, a similar number of DEGs showed that the cells may have been prepared to adapt to the low-temperature environment.

As shown in the Venn diagram (Figure 1B), at 6 h, only three genes were differentially expressed under both the 5°C and −5°C conditions; 46 genes were solely expressed at 5°C; 81 genes showed changed expression only at −5°C. After 24 h of incubation, 72 genes were differentially expressed under both the 5°C and −5°C conditions; 147 genes were specifically expressed at 5°C; 165 showed changed expression solely at −5°C. These results suggest that MS612 adopted different strategies to respond to cold stress at different time points and temperatures. There were 14 and 25 genes expressed differentially at both the 6 h and 24 h time points under the 5°C and −5°C conditions, respectively. Such common DEGs may play an important role in the regulation of the MS612 response to cold stress.

### 3.4 Functional distribution of DEGs

Using gene ontology (GO) analysis, the identified DEGs were divided according to the following domains: biological processes (BP), cellular components (CC) and molecular functions (MF) (Supplementary Table S7). During the early phase of cold stress at 5°C and −5°C, the most highly represented categories were “cellular process,” “metabolic process,” “single-organism process,” and “cellular process” in the BP domain; “cell,” “cell part,” “membrane,” “membrane part,” and “organelle” in the CC domain; “catalytic activity,” “binding,” and “transporter activity” in the MF domain (Figure 1C; Supplementary Figure S6A). The results indicated that the majority of DEGs were involved in “metabolic processes,” “cellular processes,” “cell,” “membrane components,” and “transporter activity,” suggesting that MS612 mainly resists cold stress through changes in physiological metabolism, changing membrane composition, and transport activity, as well as through cell differentiation. More intriguingly, all three categories of DEGs were more highly enriched at −5°C than 5°C, indicating that more relevant genes were highly expressed under lower temperatures to maintain basic cell functions in the early stages.

**Figure 2:**
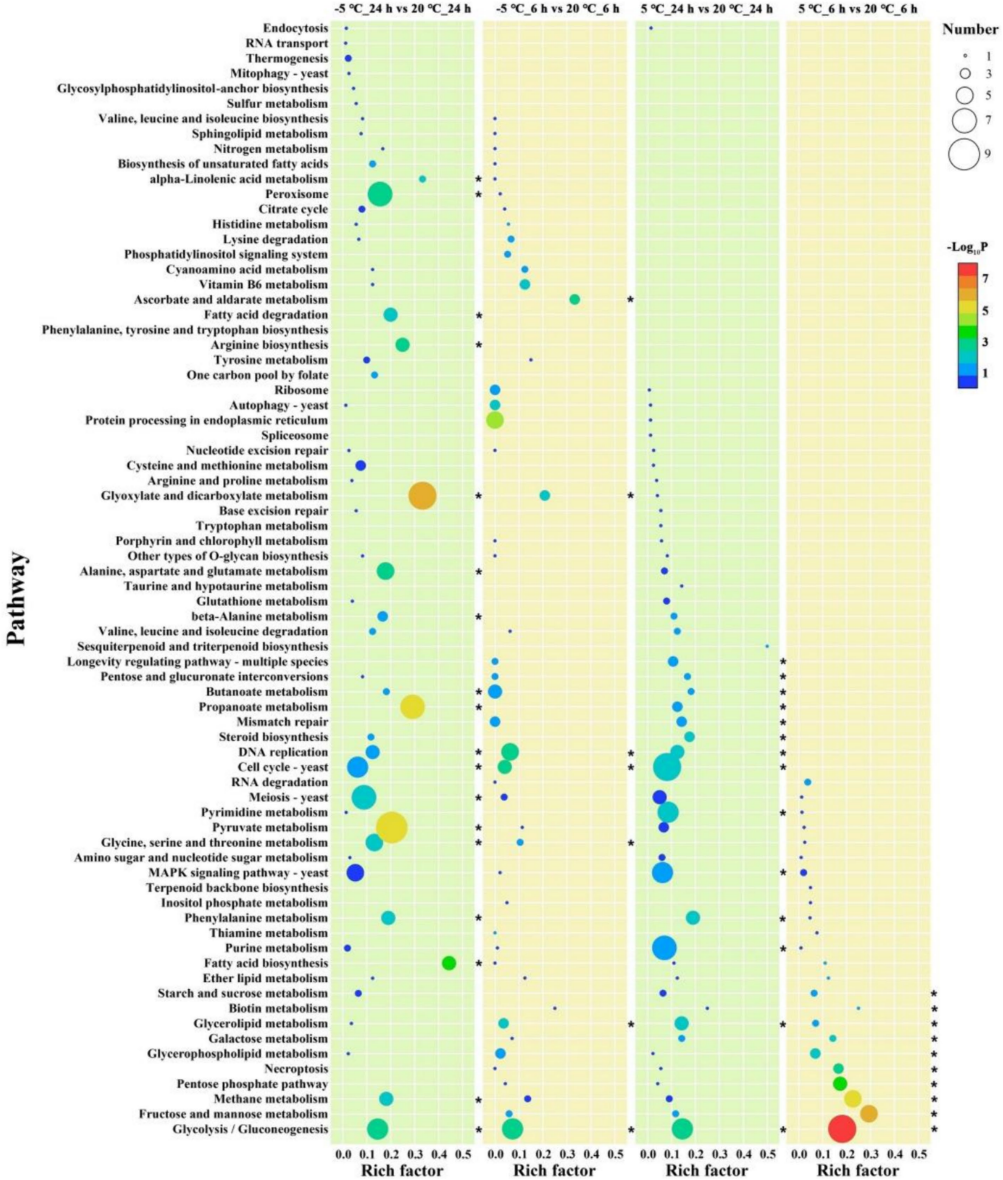
Enrichment analysis of KEGG pathway. The vertical axis represents the pathway name, and the horizontal axis represents the rich factor: the ratio of the number of genes/transcripts enriched in the pathway to the number of all annotated genes/transcripts. The larger the rich factor is, the greater the enrichment degree is; the size of the bubble indicates the number of genes in the pathway, and the color of the dot corresponds to different *p*-value ranges. ‘*’ represents *p* < 0.05.

To further identify the roles of the DEGs, we performed a KEGG pathway analysis (Supplementary Table S8). Only significantly enriched categories with *p* < 0.05 were selected, and we found that cold stress mainly affected some common pathways related to carbohydrate and energy metabolism, such as “glycolysis/gluconeogenesis,” “fructose and mannose metabolism,” and “Methane metabolism” in the early phase (Figure 2). In addition, additional pathways including “DNA replication” and “Cell cycle” showed enrichment at −5°C, indicating that lower temperatures exert pressure on cell differentiation (Figure 2).

Similar to the cold-shock phase, during the later phase of cold stress (both at 5°C and −5°C), DEGs were characterized by the GO terms “cellular process,” “metabolic process,” and “single-organism process” in the BP domain; “membrane,” “membrane part,” “cell,” “cell part,” and “organelle” in the CC domain; “catalytic activity,” “binding,” and “transporter activity” in the MF domain (Figure 1C; Supplementary Figure S6B). During the cold adaptation period, the three categories showed similar enrichment at −5°C and 5°C, indicating that “cell process,” “membrane,” and “transporter activity” remained the main factors associated with adaptations to cold stress. The KEGG enrichment analysis indicated that genes involved in more diverse pathways were activated at lower temperature treatment (Figure 1C; Supplementary Table S8). Genes involved in “Glycolysis/Gluconeogenesis,” “Phenylalanine metabolism,” “Cell cycle,” “DNA replication,” and “Propanoate metabolism” were highly enriched and observed at 5°C and −5°C, while more pathways involved in “Glyoxylate and dicarboxylate metabolism,” “Propanoate metabolism,” “Pyruvate metabolism,” “Peroxisome,” and “Alanine, aspartate and glutamate metabolism” pathways showed enrichment only at −5°C (Figure 2). Needless to say, the greater number of activated pathways was likely an outcome of low-temperature severity of the cold environment during the cold adaptation phase even though the GO analysis results did not show such differences.

### 3.5 Analysis of metabolites

The limit of a 1.000-fold change coupled with a Student’s t-test (*p* < 0.05) was used to identify the differentially expressed metabolites. The metabolic data showed that MS612 exhibited increased glycerol accumulation at 5°C. The glycerol content almost did not change during the whole freezing stress at −5°C, but the proline accumulation level was significantly increased (Table 1), the transcriptome results showed elevated expression of proline synthesis-related genes (*METSCH_C00410* and *METSCH_C00430*) (Supplementary Table S9). These metabolic data were consistent with the corresponding gene expression. At 5°C glycerol significantly enhanced cell survival by stabilizing cell membranes, resisting osmotic pressure changes and providing metabolic energy. Low temperature resulted in increased oxidative stress in cells, and proline played a significant role in redox homeostasis.

**Table 1:** Changes of proline and glycerol in MS612 under low-temperature stress at different stages (relative content).

Compound	5°C/20°C		−5°C/20°C	
	6 h	24 h	6 h	24 h
Proline	1.028167	1.439369	0.905204	2.476753
Glycerol	0.968033	1.968197	0.856223	1.107623

At Interestingly, MS612 increases the intake of iron ions under cold stress, and the absorption of these iron ions may help to resist cold stress (Supplementary Figure S7).

### 3.6 Validation of DEGs by RT-qPCR

**Figure 3:**
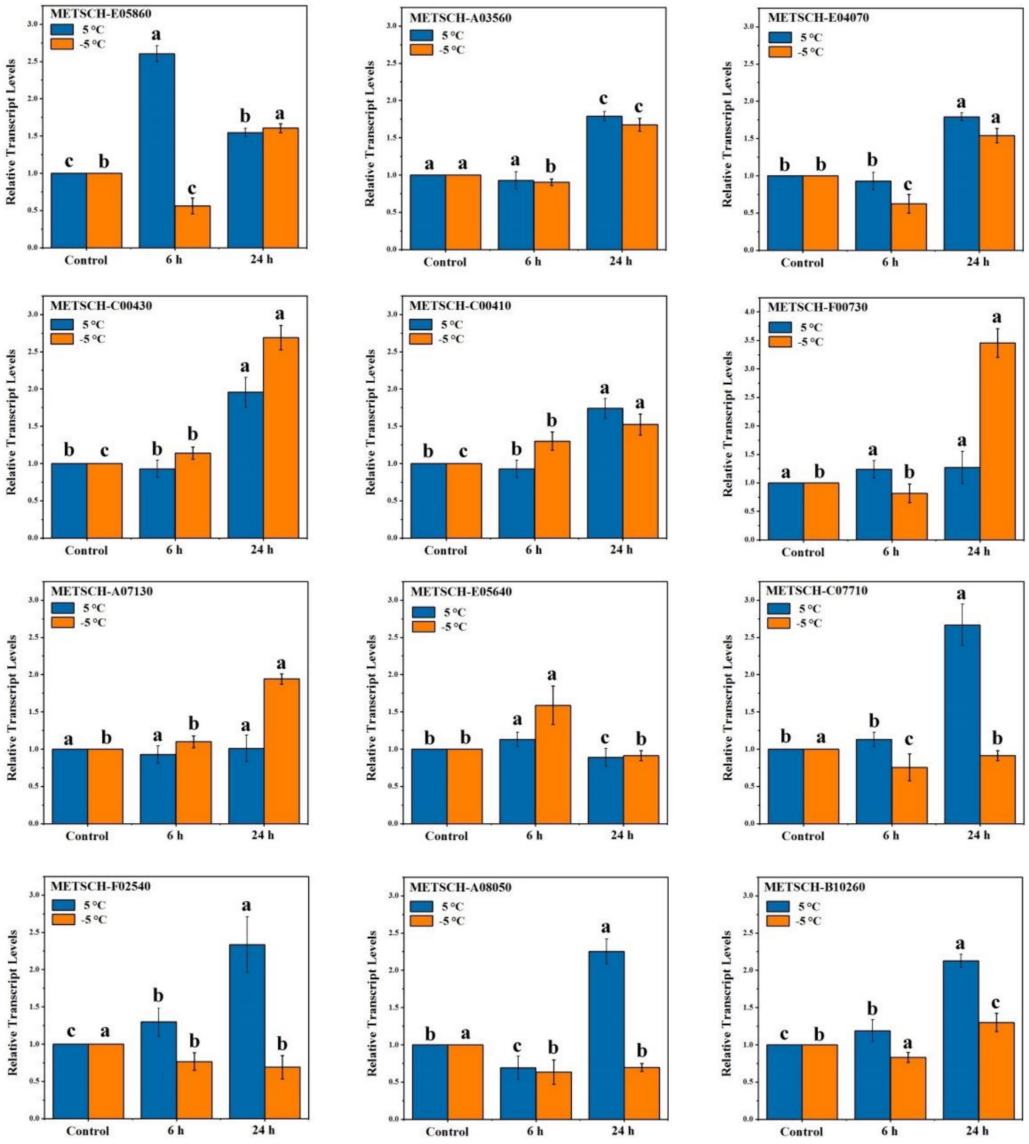
RT-qPCR validations of DEGs in response to cold stress. Statistical analyses were performed based on ANOVA, and different letters above the bars represent significance at *p* < 0.05.

To validate the expression patterns of the genes identified on comparative transcriptome analysis, we randomly selected 12 of these genes for RT-qPCR analysis using specific primers (Supplementary Table S1). The expression patterns of 10 of these 12 genes (83.3%) in MS612 were consistent with the transcriptome data. Hence, the independent evaluations confirmed the reliability of the transcriptome data (Figure 3) (Song et al., 2019).

## 4 Discussion

To better understand the potential cold tolerance mechanism of MS612, we selected 168 DEGs as genes important for cold tolerance (Supplementary Table S9). The selected candidate genes were grouped according to the above phenotypic analysis and bioinformatics analysis as follows: carbohydrate and energy metabolism, cell membrane and lipid metabolism, antifreeze metabolites, structural proteins, signal transduction and antioxidation and metal metabolism. Their expression patterns and possible roles are discussed below.

### 4.1 DEGs related to carbohydrate and energy metabolism

Metabolism is tightly related to physiological adaptations in yeasts during stress responses. Energy metabolism and carbohydrate transport are affected by cold stress (Amato and Christner, 2009). KEGG enrichment analysis showed that the expression of genes-related- to energy metabolism pathways such as glycolysis/gluconeogenesis, the citric acid cycle (TCA cycle), and the pyruvate metabolism pathway were significantly down-regulated at 5°C under cold stress (Supplementary Table S8). Previous studies have shown that ATP production is essential for cellular energy maintenance; However, ATP utilization is less necessary under cold conditions than at higher temperatures (Koh et al., 2017). Yeasts can use anaerobic fermentation to obtain energy under low temperatures, although most non-*S. cerevisiae* are intolerant to ethanol. Alcohol dehydrogenase (ADH) plays a central role in the metabolism of alcohols and aldehydes, and it is a key enzyme in anaerobic fermentation (Xu et al., 2019). Our study indicated that genes related to alcohol metabolisms, including those for short-chain alcohol dehydrogenase, alcohol dehydrogenase, and alcohol acetyltransferase, were significantly up-regulated at 5°C. Ballester-Tomás et al. (2016) found that at temperatures close to the freezing point, ADH levels were significantly up-regulated in *S. cerevisiae*. Moreover, *S. cerevisiae* cells lacking the *Adh3* gene show decreased fitness at low temperatures, while *Adh3* overexpression enhances growth under cold conditions (Paget et al., 2014). In the present study, the acetyltransferase genes (*METSCH_G00420*, *METSCH_C06340*, and *METSCH_D04130*) related to ethanol metabolism were also significantly up-regulated at 5°C under cold stress (Supplementary Table S9). Yeasts use alcohol acetyltransferase to synthesize more esters, improving wine and distillate flavor (Lilly et al., 2006). Therefore, we speculate that anaerobic fermentation is one of the main strategies used by MS612 to obtain energy at low temperatures. In the present study, the high expression of the alcohol dehydrogenase gene (*METSCH_A03560*) was maintained during cold adaptation at −5°C. We speculate that the energy maintenance strategies used by MS612 at lower temperatures may differ from those adopted under mild cold stress.

Phenylalanine decarboxylase can catalyze the decarboxylation of phenylpyruvate to phenylacetaldehyde, which is important for the participation of yeast cells in the Ehrlich pathway (Wang Y. et al., 2020). In our study, a gene encoding phenylalanine decarboxylase (*METSCH_E05860*) was specifically up-regulated in MS612 at 5°C and −5°C during the early phase of cold treatment. The results indicated that MS612 increased amino acid metabolism to obtain energy in a low-temperature environment. Vitamin B plays an important role in amino acid transfer, decarboxylation, and decomposition, and it is also involved in the metabolism of fat and carbohydrates as coenzymes (Monteverde et al., 2017). We observed the expression of genes related to vitamin B6 (*METSCH_*C03120) and biotin synthesis (*METSCH_C06160*) at −5°C during the later phase of cold treatment. Hence, the up-regulation of vitamin B-related genes may play a vital role in the ability of MS612 to resist freezing stress.

### 4.2 DEGs related to transporters

In all organisms, lipid bilayers constitute a chemical barrier against the environment and contain important ion pumps. Hence, the plasma membrane is often involved in the regulation of metal ions and metabolites, nutrient exchange, and cellular processes under low-temperature stress (Haddaji et al., 2017). Transcriptome data revealed the differential expression of transporter genes including those involved in the transport of sugars, small oligopeptides, amino acids, multidrug resistance proteins, and metal ions during cold stress in MS612 (Table 2; Supplementary Table S9). The Major Facilitator Superfamily (MFS) is a major secondary membrane transporter superfamily, and it is the largest known secondary carrier superfamily in the biosphere (Yan, 2013). Our study indicated that most genes from the MFS, especially those involved in sugar transport, were up-regulated at 5°C (Table 2; Supplementary Table S9). Members of the sugar transporter (SP) subfamily are vital for metabolism and energy homeostasis in bacteria, archaea, fungi, protozoa, plants, and animals because they mediate the cellular uptake of glucose and other mono- and disaccharides (Henderson and Baldwin, 2012). However, most sugar transport-related genes were down-regulated at −5°C. After 24 h of cold treatment, only three out of 13 sugar transport-related genes (*METSCH_E04070*, *METSCH_D02420* and *METSCH_E06280*) were up-regulated at −5°C, although more were up-regulated at 5°C (Supplementary Table S9). Therefore, the sugar transport system could form an important part of the survival strategy of cold-tolerant yeasts during cold adaptation at 5°C. On the contrary, more amino acid transporter genes were up-regulated at −5°C than at 5°C during the later phase. The accumulation of specific amino acids and secondary metabolites produced via amino acid metabolism has been hypothesized to increase tolerance to adverse environmental conditions (Ting et al., 2010). Our results revealed that nitrogen source-related transport genes may be essential for freezing stress adaptation. Further, amino acids may act as the main energy source and provide sufficient energy to allow MS612 to overcome cold stress.

**Table 2:** Comparative transcriptomic analysis of transporter genes at different time points during the cultivation of MS612 under cold stress.

Gene ID	5°C/20°C				−5°C/20°C			
	6 h		24 h		6 h		24 h	
	Log_2_ FC	*p*-adjust	Log_2_ FC	*p*-adjust	Log_2_ FC	*p*-adjust	Log_2_ FC	*p*-adjust
Sugar transporter								
METSCH_A02240	1.328	0.801	**1.969**	**0.000**	**−3.931**	**0.006**	**1.893**	**0.052**
METSCH_E06280	0.635	0.774	**1.797**	**0.000**	−0.723	0.519	0.882	0.004
METSCH_C06370	2.716	0.446	**2.522**	**0.024**	**−2.797**	**0.002**	−0.241	0.783
METSCH_E04070	2.226	0.319	**1.353**	**0.000**	−2.425	0.126	**1.055**	**0.025**
METSCH_G00470	0.283	0.911	**1.241**	**0.016**	−0.351	0.962	0.920	0.125
Drug resistance transporter								
METSCH_A06580	0.099	0.927	−0.204	0.369	**1.197**	**0.046**	−0.235	0.119
METSCH_D00780	−0.366	0.488	**1.047**	**0.026**	0.624	0.664	0.246	0.388
METSCH_A01980	−0.101	0.977	**1.265**	**0.000**	0.960	0.373	**2.337**	**0.000**
METSCH_F01030	0.510	0.848	0.517	0.312	1.339	0.398	**1.744**	**0.001**
METSCH_G02260	2.287	0.640	1.781	0.347	1.696	1.000	**2.736**	**0.041**
METSCH_A03850	1.487	0.126	**1.602**	**0.000**	−0.453	0.818	0.805	0.051
Amino acid transporter								
METSCH_C00120	0.803	0.212	**1.494**	**0.000**	−0.436	0.916	**2.361**	**0.000**
METSCH_A05520	−0.455	0.825	−0.546	0.052	−0.729	0.353	**−1.121**	**0.000**
METSCH_F03770	−0.072	0.983	**−1.349**	**0.000**	−0.949	0.313	−0.773	0.001
METSCH_A12560	0.568	0.459	0.492	0.019	−0.553	0.659	0.467	0.036
METSCH_B05610	**−1.599**	**0.112**	0.667	0.165	−0.334	0.952	0.113	0.802
METSCH_E00900	0.136	0.979	0.073	0.904	1.631	0.176	**2.295**	**0.000**
METSCH_C00410	−0.214	0.917	**1.033**	**0.000**	−0.141	0.991	0.764	0.003
METSCH_C00430	1.473	0.397	**1.799**	**0.012**	−1.551	0.263	**1.094**	**0.193**
High-affinity iron transporter								
METSCH_F00730	**2.126**	**0.000**	1.905	0.093	−0.880	0.844	**2.503**	**0.004**
METSCH_E02540	0.378	0.369	**1.280**	**0.016**	−1.163	0.991	0.814	0.044

Log_2_FC is Log_2_ Fold Change. Positive value means up-regulated and negative value means down-regulated. A *p*-adjusted value of less than 0.05 indicated significance, and a *p*-adjusted value of less than 0.001 indicated extreme significance. The bolded font indicates genes with significant changes associated with transporter proteins at different time points.

It is also worth noting that genes for multidrug resistance (MDR) protein and metal ion (especially ferric ion) transporters were also differentially expressed under cold stress. Several studies have demonstrated that MDR transport genes are strictly regulated, and their differential expression indicates the presence of potentially toxic substances (including ethanol, ROS, and toxins) in the intracellular and extracellular environment (Nelissen et al., 2019). Our study shows that the expression of MDR-related transport genes is higher at −5°C than at 5°C (Supplementary Table S9). We speculate that Under lower temperatures (−5°C), owing to the accelerated accumulation of cellular endotoxins, rapid toxin removal promotes cell survival. Iron is necessary for fungal growth and pathogen reproduction. Iron metabolism has been well characterized in the *S. cerevisiae* model (Prevorovský et al., 2009; Sadineni et al., 2012). Previous reports show that the *M. pulcherrima* yeast can inhibit Botrytis cinerea, Alternaria, and Staphylococcus expansins through iron competition (Saravanakumar et al., 2008). It is essential to maintain the homeostasis of metal ions under biological and abiotic stress. Loosely bound iron is not properly regulated by normal metal transport and storage mechanisms in the cell, resulting in increased oxidative stress (Zecca et al., 2004; Kim et al., 2021). Herein, we found that several genes related to iron metabolism, including those for ferric reductase (*METSCH_B03390* and *METSCH_E00190*) and high-affinity iron transporters (*METSCH_F00730* and *METSCH_E02540*), were significantly up-regulated under cold stress (Table 2; Supplementary Table S9). The steady-state of intracellular metal ions is necessary at low temperatures, but the mechanisms by which these iron transport genes are regulated remain to be studied in the future.

### 4.3 DEGs related to the cell membrane, lipid metabolism and cell wall

Changes in ambient temperature are first sensed by the cell membrane. Low temperatures can reduce membrane fluidity, make the membrane harder, and lead to biological dysfunction of the membrane (Chen et al., 2022). Cell membranes are composed of several different lipid and sterol products, including phospholipids, glycolipids, sphingolipids, various proteins, and sterols (Valitova et al., 2019). By changing the phospholipid head group, sterol content, and short-chain fatty acid composition and by reducing the degree of unsaturation in fatty acids, the membrane’s fluidity index can be maintained despite changes in environmental temperatures (Rodríguez-Vargas et al., 2007). Previous studies have confirmed the key role of fatty acid desaturase in cold stress (Chen et al., 2022). In our study, DEGs related to fatty acid desaturation were not identified in our study. However, genes related to lipid oxidation (*METSCH_B04870* and *METSCH_B04860*) were found to be significantly down-regulated at −5°C (Supplementary Table S9). It is worth noting that the composition of sterols plays an important role in regulating membrane properties, including fluidity. Higher sterol levels increase a cell’s tolerance to extreme temperatures and ethanol (Beney and Gervais, 2001). We found 13 DEGs related to sterol metabolism, the Delta (24(24(1)))-sterol reductase (*METSCH_C00980*), C-8 sterol isomerase (*METSCH_E05640*), C-4 methylsterol oxidase (*METSCH_A07130*), and lathosterol oxidase (*METSCH_A13320*) genes were differentially expressed throughout the cold treatment period (Supplementary Table S9). These results suggest that the up-regulation of sterol-related genes may be the main strategy for maintaining membrane fluidity in MS612 under severe cold stress.

The cell wall is the most important outer structure in cells. Cells can modify the structure of the cell wall in response to environmental challenges (Sanz et al., 2012). *β*-1,3-glucan is the skeleton structure in the cell wall and the main scaffold of cell wall proteins (Nerome et al., 2021). In this study, one glucan 1,3-beta-glucosidase gene (*METSCH_B10030*) was up-regulated at 5°C during the later phase, although its expression appeared unchanged at −5°C. At 5°C, the cell wall in yeast may be slightly degraded to cope with low temperature environment, but at −5°C, the expression has no significant change, indicating that temperature is not positively related to the expression of glucan 1,3-beta glucosidase gene. In addition, the deacetylation of chitin is also important for chitosan synthesis and cell wall recycling (Zakrzewska et al., 2005; Wei et al., 2021). Our study also revealed the up-regulation of one chitin deacetylase gene (*METSCH_G01800*) at −5°C. The increase in chitin deacetylation may lead to cell wall thickening, protecting cells from freezing and osmotic pressure disruption at low temperatures. Cold stress may be increased the degradation of cell wall, but the increased synthesis of chitosan keeps the integrity of cell wall to attenuate cold stress.

### 4.4 DEGs related to antifreeze metabolites and structural proteins

Metabolic adaptation is indispensable for abiotic stress tolerance in cells. To reduce cold stress injuries, cells secrete several antifreeze agents, such as glycerin and trehalose (Sun et al., 2020). Glycerin-3-phosphate dehydrogenase (GPD) is a major enzyme for glycerol synthesis, and mutants lacking this gene do not produce any glycerol. As a classic substance secreted under low-temperature stress, glycerol has an osmotic effect, reducing the inner osmotic pressure and the freezing point of cells (Albertyn et al., 1994). In this study, two GPD genes (*METSCH_A08050* and *METSCH_B10260*) we were found to be significantly up-regulated in the cold adaptation stage at 5°C (Supplementary Table S9). In contrast, these genes related to glycerol secretion were not further differentially expressed at −5°C. Although genes (*METSCH_B09780*) related to starch metabolism were up-regulated at 5°C, genes for other classic antifreeze agents such as trehalose and glycogen were not overexpressed under any conditions of cold stress. These findings were consistent with those from Wei et al. (2021). We speculate that the enzymes responsible for the synthesis of key compounds or intermediates are inhibited by low temperatures. Hence, the yeast changes its metabolic strategy and uses other substances as antifreeze protectors under freezing stress.

Misfolded proteins accumulate low temperatures in cryophylactic organisms. To ensure that proteins are folded appropriately, cells up-regulate molecular chaperones to prevent hypothermia-induced protein misfolding (Sherman and Goldberg, 1996). Studies in bacteria and yeast have shown that the regulatory members of the CLP family (mainly ClpA, ClpB, and ClpC) and their catalytic subunits (mainly ClpP) form an ATP-dependent two-component protein hydrolysis system (Lee et al., 2007). This system not only participates in the regulation and control of energy-dependent protein hydrolysis but also functions as a molecular chaperone (Lee et al., 2007). In our study, we observed that MS612 up-regulated the ATP-dependent Clp protease ATP-binding subunit ClpB-related gene (*METSCH_C04540*) to degrade misfolded proteins after 24 h of cold treatment at 5°C (Supplementary Table S9). Similarly, one cyclophilin-related gene (*METSCH_A02810*) associated with protein folding was significantly down-regulated in the later stage of cold stress, suggesting that it may have participated in protein folding during the early stage (Jung et al., 2020).

The enhancement of amino acids under low temperatures indicates that the metabolic strategy of yeast cells under freezing conditions differs from that under cold stress. Proline, as a compatible osmolyte, has a variety of adaptive functions under stress, including membrane and protein stabilization, free radical scavenging, and the provision of carbon, nitrogen, and energy for post-stress recovery (Hoffmann and Bremer, 2011). In our study, two proline transport-related genes (*METSCH_C00410* and *METSCH_C00430*) were significantly up-regulated during the cold adaptation period in MS612 (Supplementary Table S9). This indicates that the expression of genes regulating the accumulation of compatible solutes such as proline and glycerin helps cells overcome the osmotic contraction caused by intracellular freezing and thawing and promotes survival in a low-temperature environment (Panadero et al., 2006).

Interestingly, transporter genes related to oligopeptides and proline were further up-regulated at −5°C. We infer that the protein hydrolysis system can hydrolyze the accumulated misfolded proteins into carbon and nitrogen sources, because molecular chaperones may not be effective in nutrient-limited sub-zero temperatures. These proteolytic systems can also promote the accumulation of intracellular amino acids, providing reservoirs for the synthesis of specific proteins as well as a source of facultative solutes (Koh et al., 2017). The decrease in proteolytic enzyme activity further leads to the accumulation of unfolded proteins in the ER membrane. Hence, improving the transport of these unfolded proteins could help alleviate the pressure in the ER under freezing temperatures.

### 4.5 DEGs related to signal transduction

**Figure 4:**
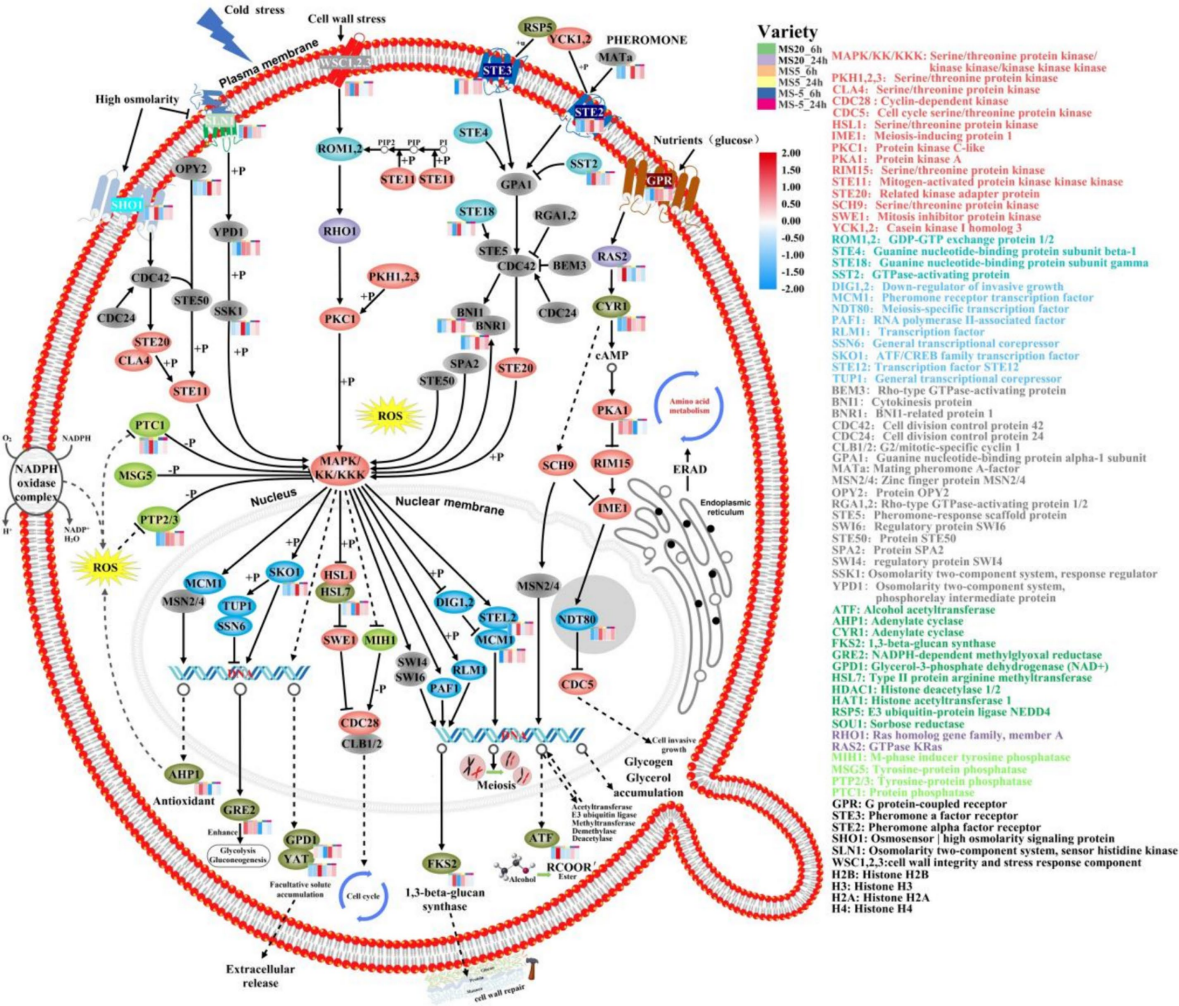
Analysis of main signaling pathways of MS612 under cold stress based on KEGG. The colors of the ellipses are gray represents a protein, red represents kinase, green represents phosphatase, blue represents transcription factor, dark green represents enzyme, purple represents GTP enzyme, and pink represents G protein. The bar chart in the upper right corner represents different conditions for MS612: green indicates incubation at 20°C for 6 h, purple for 24 h; orange for 6 h at 5°C, yellow for 24 h; blue for 6 h at −5°C, and red for 24 h.

Signaling pathways are capable the cell of monitoring external and internal states and to express the suitable physiological responses (Lee and Yaffe, 2016). KEGG analysis showed that the mitogen-activated protein kinase (MAPK) signaling pathway, which is involved in apoptosis, was significantly enriched in the late stage during low-temperature stress (Figure 4). Our study found that five serine/threonine protein kinase related genes were differentially regulated in MS612 at 5°C during the later phase of cold treatment; four genes (*METSCH_C07960*, *METSCH_C01570*, *METSCH_F00410*, and *METSCH_D01040*) were down-regulated, while one (*METSCH_A07380*) was up-regulated (Supplementary Table S9). Apoptosis is a precisely controlled physiological process that is necessary for coping with stress and infection and for maintaining homeostasis. Apoptosis is regulated by signaling pathways that involve protein-tyrosine phosphatase (PTPs) (Hallé et al., 2007). The identified PTP-related genes (*METSCH_C07710* and *METSCH_F02540*) were found to be significantly up-regulated during the cold adaptation phase at 5°C in our study (Supplementary Table S9). These PTPs act as negative regulators of serine/threonine-protein kinases in the MAPK pathway and thus inactivate the cell death pathway triggered by the serine/threonine protein kinase (Ghosh et al., 2014). Previous reports have shown that PTPs can prevent the lethal effects induced by the overexpression of Hog1-a protein involved in glycerol synthesis in *S. cerevisiae* and block inappropriate cross-talk between the HOG pathway and the cell-wall integrity MAPK pathways (Dunayevich et al., 2018). This indicates that PTPs are important for maintaining specificity in MAPK signaling pathways (Winkler et al., 2002). The related signal transduction elements Swe1 (*METSCH_C07410*) and Hsl1 (*METSCH_C07960*) were also found to be significantly down-regulated in our study, further confirming that serine/threonine protein kinases were inhibited at 5°C (Supplementary Table S9). In addition, G protein-coupled receptor (Gpr1), a cell membrane component regulated by environmental nutrients (glucose), was also up-regulated, further promoting the protein kinase A system (cAMP-PKA) pathway and triggering cell invasion (Lemaire et al., 2004). Similarly, the cAMP-PKA pathway could further inhibit a serine/threonine-protein kinase (rim15) (Granek et al., 2010). The differential expression of genes related to these signaling pathways partially explains how MS612 growth is not inhibited near the freezing point (0–5°C).

At below-zero temperatures (−5°C), the growth rate of cells was significantly reduced (Supplementary Figure S4). MS612 down-regulated mitosis-related genes (Clb1/2 and MCM2/4) and up-regulated the pheromone cAMP-PKA pathway and meiosis-related genes (Gpa1, Ste5, Ama1, Rec8, and Ume6) (Supplementary Table S9). The down-regulation of Cdc5 (cell cycle serine/threonine protein kinase) and PTPs indicated that the weak cells had all died or become inactive. Further, it also suggested that the cAMP-PKA signaling pathway may no longer be involved in the regulation of cell growth under freezing temperatures.

ERAD mediates the turnover of short-lived and misfolded proteins in the ER membrane or lumen. Maintaining protein homeostasis, especially in the ER, is very challenging under low temperatures because the high demand for protein synthesis constantly leads to misfolding stress (Haas, 2014). To counteract the disastrous effects associated with the accumulation of defective proteins, misfolded ER proteins are targeted and degraded via ERAD. Loertscher et al. showed that ERAD plays a role in cold adaptation, perhaps through effects on sterol biosynthesis (Loertscher et al., 2006). In our study, the genes related to the transport and metabolism of amino acids, small oligopeptides, and proteins are further up-regulated at freezing temperatures. However, protein hydrolysis-related genes were not differentially expressed at −5°C. We hypothesized that proteolytic enzymes have reduced reduce their catalytic activity due to freezing temperatures. Alternatively, too many misfolded proteins remained unhydrolyzed in the ER, and resulting in excessive internal ER membrane pressure. Obviously, improving the transport of amino acids and proteins in the ER membrane could mitigate this problem. In addition, the high expression of sterols mentioned previously is closely related to the ERAD signaling pathway under freezing conditions.

## 5 Conclusion

In this study, comparative transcriptome analysis was performed to investigate the potential tolerance mechanisms of MS612 under cold stress. The transcriptomic analysis of MS612 showed that many metabolic pathways were affected under different temperature of cold stress. When the temperature drops to 5°C, MS612 enhanced low temperature adaptability through increasing energy metabolism, sugar transport, increasing sterol lipid synthesis, glycerol synthesis, degrading misfolding protein, inhibiting cell death pathway and carrying out antioxidant pathway. While, when the temperature is further reduced to −5°C (below freezing point), MS612 increased the metabolism related to cell cycle, cell meiosis, lipid synthesis, proline transport and metal homeostasis pathways. The findings suggest that under low-temperature and freezing stress, this cryophylactic yeast undergoes physiological adaptations by altering its main signaling transduction patterns. This leads to changes in energy metabolism, substance transport, antifreeze protection, and other processes. Overall, our findings provide valuable insights into the strategies of cold adaptation in yeast.

## Data Availability

The original contributions presented in the study are included in the article/Supplementary material, further inquiries can be directed to the corresponding authors.
