# Supplementary material for: Investigation of cold-resistance mechanisms in cryophylactic yeast Metschnikowia pulcherrima based on comparative transcriptome analysis
Source: Front Microbiol. 2024 Sep 25;15:1476087. doi: 10.3389/fmicb.2024.1476087 (PMC11462854; doi:10.3389/fmicb.2024.1476087)
Supplement: Supplementary file 1 [file Table_1.DOCX]

Supplementary Material

Catalog

[Supplementary Figures 1](#_Toc105)

[Figure S1. Preliminary isolation of target fungi](#_Toc5564) *[M. Pulcherrima](#_Toc5564)* [MS612 (23/24A). 1](#_Toc5564)

[Figure S2. Cell sample information of](#_Toc30846) *[M. pulcherrima](#_Toc30846)* [MS612(MS612). 2](#_Toc30846)

[Figure S3. Flow chart of experiment design. 3](#_Toc5112)

[Figure S4. The growth curve of the strain at different temperatures. 4](#_Toc12919)

[Figure S5. Yeast growth curve. 5](#_Toc4006)

[Figure S6. Function classification of the DEGs. 6](#_Toc27591)

[Figure S7. Changes of the medium iron content (added 10 µg/mL FeSO](#_Toc25421)_[4](#_Toc25421)_[) were determined at different time points during the cultivation of MS612 7](#_Toc25421)

[Supplementary Tables 8](#_Toc25647)

[Table S1. RT-qPCR primers and amplification information for reference and target genes. 8](#_Toc18842)

[Table S2. The growth parameters (µ](#_Toc17877)_[max](#_Toc17877)_ [and AUC) of MS612 and Top15 in 84 h at different temperatures 9](#_Toc17877)

[Table S3. Summary of transcriptome sequencing 10](#_Toc23970)

[Table S4. The expressed genes and transcripts were analyzed by functional database annotation (NR, Swiss-Prot, Pfam, COG, GO and KEGG). 11](#_Toc31476)

[Additional file 1: Table S5. Identified genes for MS612 by comparative transcriptome technology. 11](#_Toc5698)

[Additional file 2: Table S6. Identification of differentially expressed genes in MS612 at different temperatures and time points. 11](#_Toc17982)

[Additional file 3: Table S7. Gene Ontology of proteins (GO) annotation of differentially expressed genes in MS612 at different temperatures and time points. 11](#_Toc23941)

[Additional file 4: Table S8. Kyoto Encyclopedia of Genes and Genomes (KEGG) annotation of differentially expressed genes in MS612 at different temperatures and time points. 11](#_Toc23340)

[Additional file 5: Table S9. The list of putative candidate genes after cold stress in MS612. 11](#_Toc11320)

# Supplementary Figures


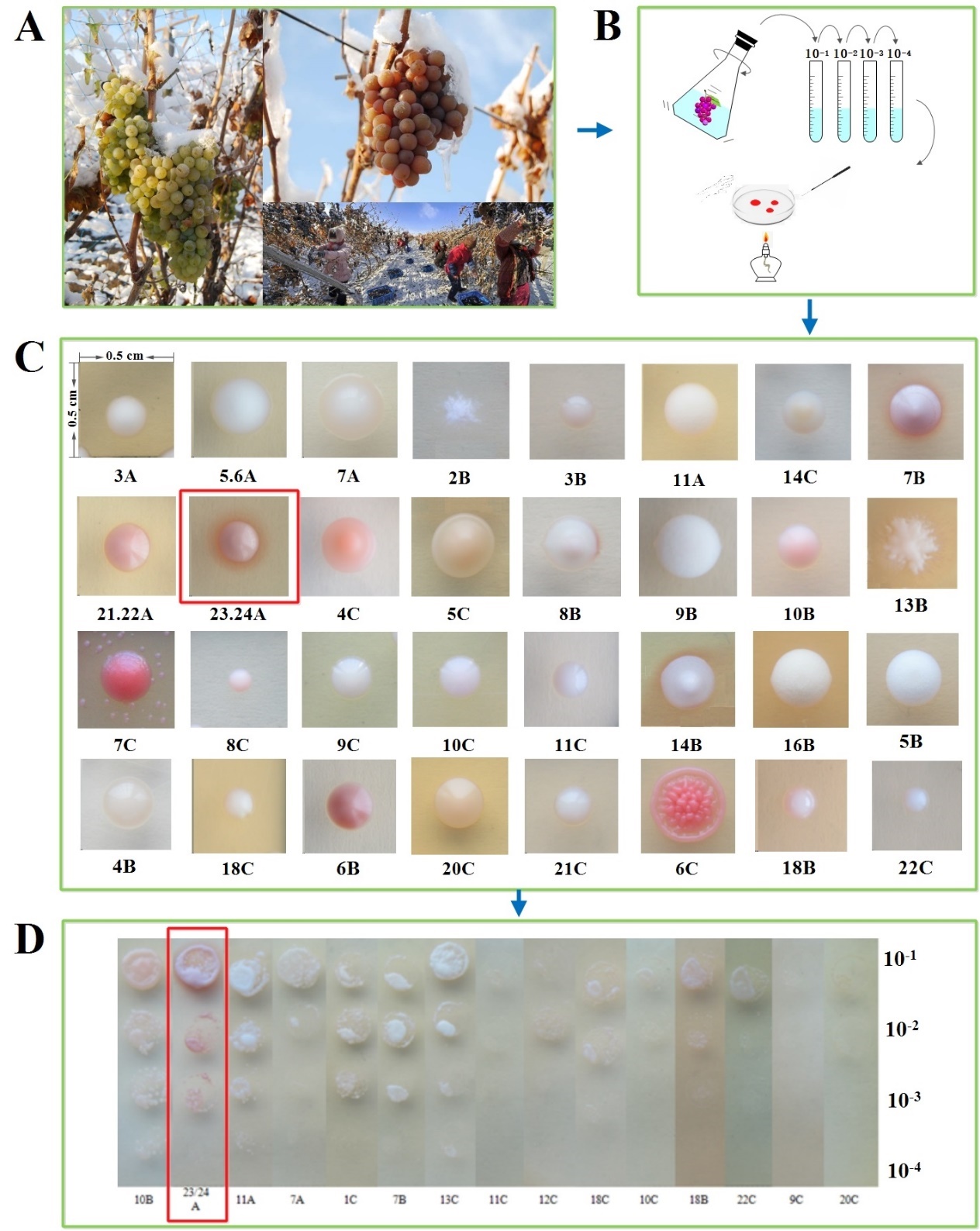


Figure S1. Preliminary isolation of target fungi *M. Pulcherrima* MS612 (23/24A). **(A)** The Vidal ice grapes; **(B)** Isolation of grape epidermal microorganisms; **(C)** Fungal colonies in the separable part of grape epidermis; **(D)** Comparative growth experiment (5 ℃) of selecting fungi with low-temperature growth potential.


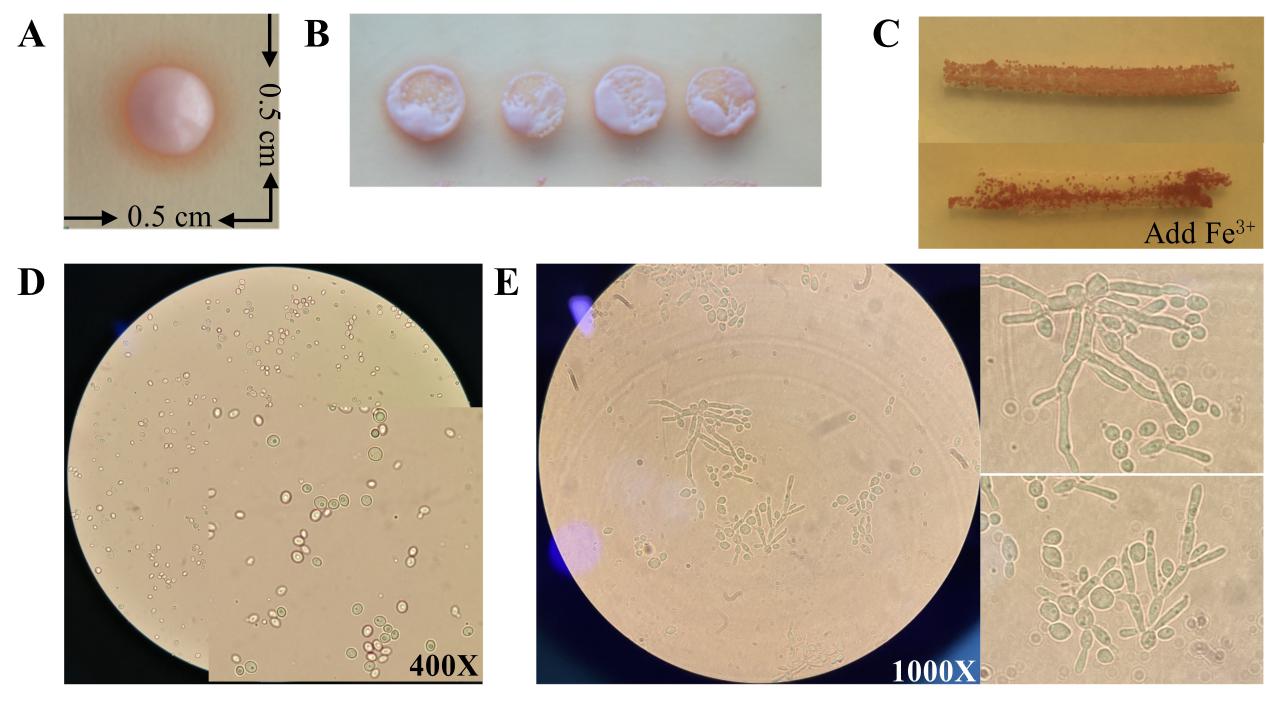


Figure S2. Cell sample information of *M. pulcherrima* MS612(MS612). **(A)** Cell colony; **(B)** Typical extracellular pigment; **(C)** The red color of cells was further deepened in YPD medium added iron ions; **(D)** The cell morphology at 5 ℃; **(E)** The cell morphology at -5 ℃.


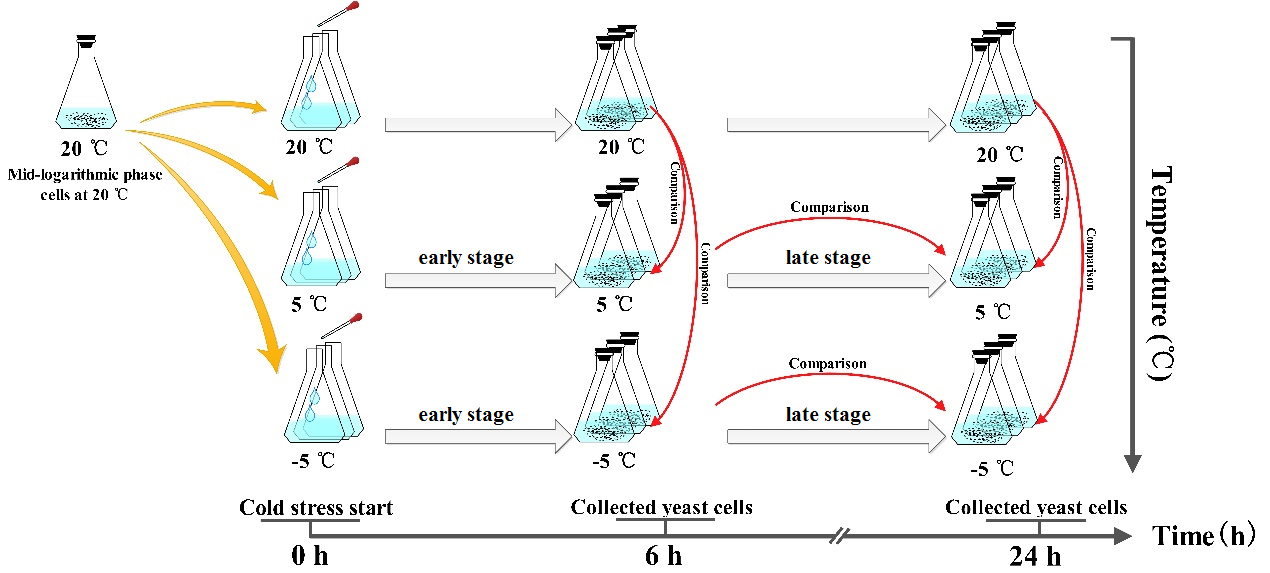


## Figure S3. Flow chart of experiment design.


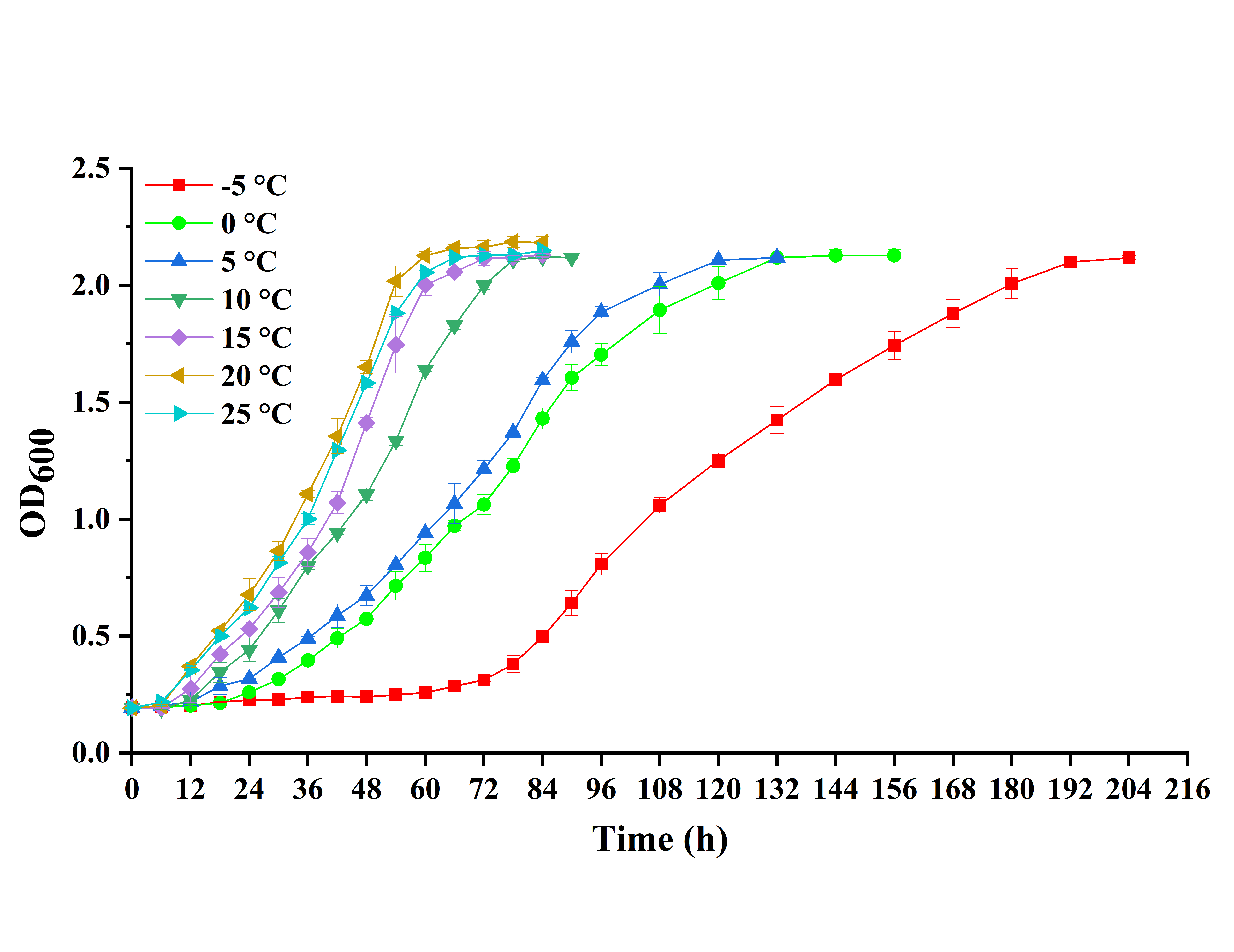


Figure S4. The growth curve of the strain at different temperatures. Growth curves of MS612 at 7 temperatures (-5 ℃, 0 ℃, 5 ℃, 10 ℃, 15 ℃, 20 ℃, 25 ℃); Cells were cultured in YPD, and the OD_600_ values were measured spectrophotometrically.


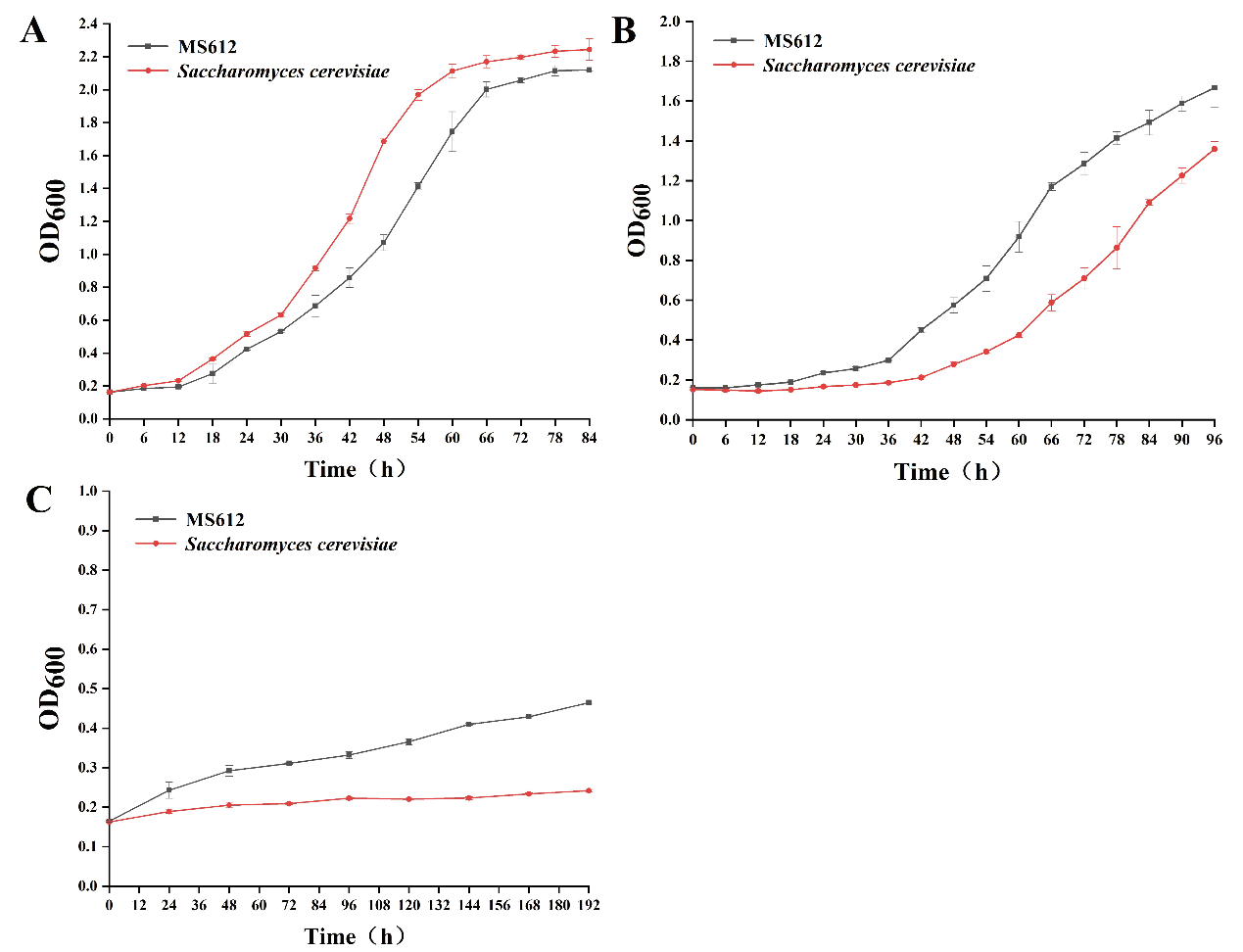


Figure S5. Yeast growth curve. **(A)** The growth curve of MS612 and *S. cerevisiae* Top15 (Top15) at 20 °C; **(B)** The growth curve of MS612 and Top15 at 5 °C; **(C)** The growth curve of MS612 and Top15 at -5 °C. *S. cerevisiae* Top15 (special yeast for ice wine low-temperature fermentation) was used as growth control.


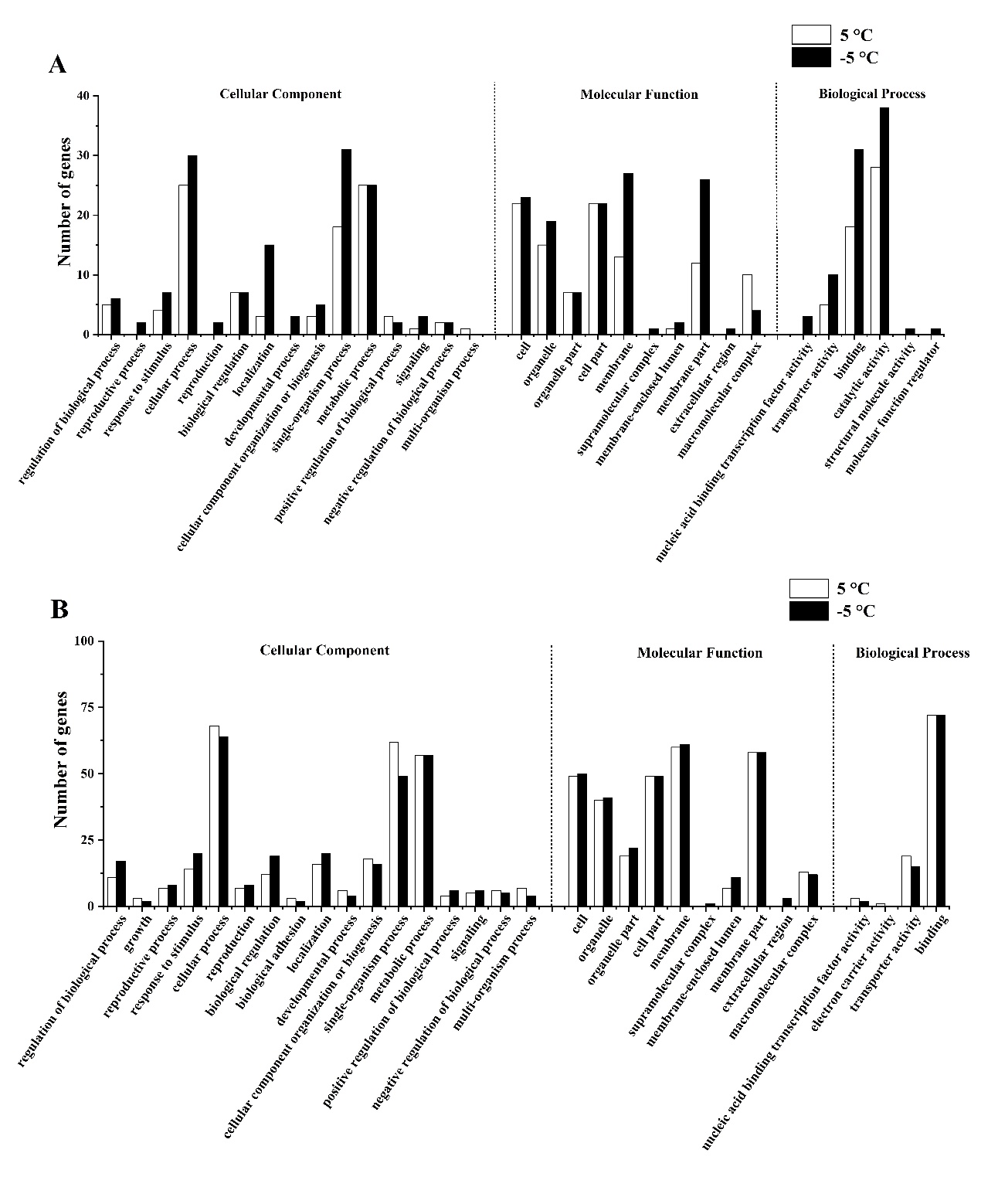


Figure S6. Function classification of the DEGs. **(A)** GO annotation of DEGs in MS612 during the early phase (6 h) of cold treatment; **(B)** GO annotation of DEGs in MS612 during the later phase (24 h) of cold treatment.


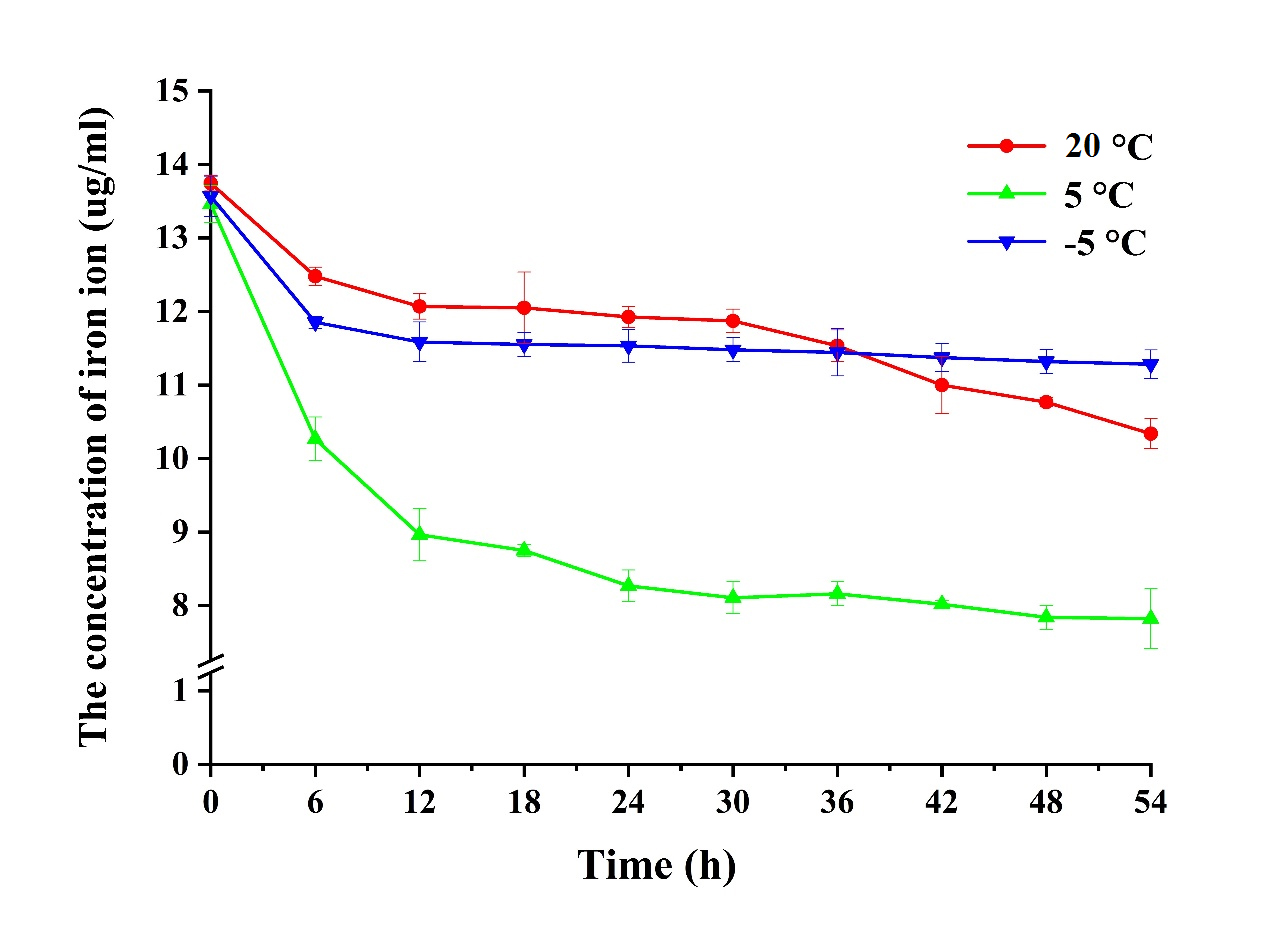


## Figure S7. Changes of the medium iron content (added 10 µg/mL FeSO_4_) were determined at different time points during the cultivation of MS612

# Supplementary Tables

## Table S1. RT-qPCR primers and amplification information for reference and target genes.

| Number | Reference/Target gene | Description | Primer Sequence (5'-3') | Product length (bp) |
| --- | --- | --- | --- | --- |
| 1 | METSCH_C02150 | actin-related protein 2 | F: GCTCCTCGATGTATCCTGGC  R: TATCCAACCGATCTGCGTCG | 99 |
| 2 | METSCH_E05860 | phenylpyruvate decarboxylase | F: ACTTTCCGCCGTAAACACCA  R: CTCTGTTCTCGGTGATGCGT | 164 |
| 3 | METSCH_A03560 | alcohol dehydrogenase NADP+ | F: GGCCATTATAGGTCGCCACA  R: TCGGGCTCGTGTAATGAGTG | 82 |
| 4 | METSCH_E04070 | MFS transporter, SP family, general alpha glucoside:H+ symporter | F: GGAAGACTTCACAGTGCCGA  R: GGAACCCTCTTGTCGTGGTT | 123 |
| 5 | METSCH_C00430 | proline transporter, YAT family | F: CGAGAAGGAGCCTGACACAG  R: AATTCCGGACGCCCTTCTTT | 84 |
| 6 | METSCH_C00410 | proline transporter, YAT family | F: TCCACCGTCAACAGATTCGG  R: CTTCGGTGGAAGAGGACGAC | 100 |
| 7 | METSCH_F00730 | high-affinity iron transporter | F: TTCGCTGATGATAAGCCCCC  R: ATGGCGAATTGGTGAAACGC | 137 |
| 8 | METSCH_A07130 | C-4 methylsterol oxidase | F: CTCAGCAGCCATGCCAAATG  R: ATGGCACTACTGGTTGCACA | 103 |
| 9 | METSCH_E05640 | C-8 sterol isomerase | F: AGTCCGGTAGAACGAGTGGA  R: AGGTCACGTGAAGCAGTACG | 139 |
| 10 | METSCH_C07710 | protein-tyrosine phosphatase | F: TCAAGACCGGACAACACTCG  R: ATGCTGCACACTTCGGGTAA | 131 |
| 11 | METSCH_F02540 | protein-tyrosine phosphatase | F: GCCTCTGTACCTGAATGCGT  R: ACGAGGTGTTGGCCATGATT | 158 |
| 12 | METSCH_A08050 | glycerol-3-phosphate dehydrogenase NAD+ | F: AGCAGAAAGACCCTGGATGC  R: TTCGTCGAAGGTGAGGGTTG | 120 |
| 13 | METSCH_B10260 | glycerol-3-phosphate dehydrogenase NAD+ | F: CTTCTTTGGCCGTGAGGACA  R: GGTCGTAACGTCAGAGTGGG | 115 |

## Table S2. The growth parameters (µ_max_ and AUC) of MS612 and Top15 in 84 h at different temperatures

| Temperature (°C) | µ_max_ (10*h^-1^) | | AUC | |
| --- | --- | --- | --- | --- |
|  | MS612 | Top15 | MS612 | Top15 |
| 25 | 0.417 | - | 117.15 | - |
| 20 | 0.463 | - | 107.00 | - |
| 15 | 0.452 | 0.664 | 99.91 | 105.91 |
| 10 | 0.451 | - | 88.40 | - |
| 5 | 0.427 | 0.541 | 56.63 | 30.07 |
| 0 | 0.378 | - | 48.34 | - |
| -5 | 0.325 | 0.223 | 21.77 | 15.36 |

*The data was calculated using the mean value of biological replication.* *“**-” means the data is not measured.*

## Table S3. Summary of transcriptome sequencing

| Sample | Raw reads | Clean reads | Error rate (%) | Q20 (%) | Q30 (%) | GC content (%) | Notes |
| --- | --- | --- | --- | --- | --- | --- | --- |
| MS20 ℃_24 h_1 | 47133534 | 46891074 | 0.023 | 98.82 | 96.26 | 50.09 | Replicate 1 |
| MS20 ℃_24 h_2 | 49408922 | 49157476 | 0.0232 | 98.72 | 95.98 | 50.18 | Replicate 2 |
| MS20 ℃_24 h_3 | 47103298 | 46820584 | 0.0236 | 98.55 | 95.54 | 50.14 | Replicate 3 |
| MS20 ℃_6 h_1 | 48286852 | 48000346 | 0.0237 | 98.53 | 95.46 | 50.24 | Replicate 1 |
| MS20 ℃_6 h_2 | 48332150 | 48069688 | 0.0233 | 98.69 | 95.89 | 50.23 | Replicate 2 |
| MS20 ℃_6 h_3 | 44746710 | 44483678 | 0.0235 | 98.62 | 95.73 | 50.28 | Replicate 3 |
| MS5 ℃_24 h_1 | 46693632 | 46285978 | 0.0234 | 98.63 | 95.77 | 50.04 | Replicate 1 |
| MS5 ℃_24 h_2 | 51062224 | 50800326 | 0.0232 | 98.73 | 95.99 | 50.05 | Replicate 2 |
| MS5 ℃_24 h_3 | 43699974 | 43434304 | 0.0232 | 98.76 | 96.07 | 50.07 | Replicate 3 |
| MS5 ℃_6 h_1 | 45454314 | 45133562 | 0.0237 | 98.54 | 95.5 | 50.05 | Replicate 1 |
| MS5 ℃_6 h_2 | 51354116 | 51077366 | 0.0233 | 98.7 | 95.9 | 49.92 | Replicate 2 |
| MS5 ℃_6 h_3 | 46426790 | 46171866 | 0.0236 | 98.59 | 95.62 | 50.03 | Replicate 3 |
| MS-5 ℃_24 h_1 | 51022038 | 50762124 | 0.0231 | 98.76 | 96.08 | 50.29 | Replicate 1 |
| MS-5 ℃_24 h_2 | 47214602 | 46981898 | 0.023 | 98.84 | 96.28 | 50.09 | Replicate 2 |
| MS-5 ℃_24 h_3 | 45072838 | 44840738 | 0.0231 | 98.78 | 96.16 | 50.1 | Replicate 3 |
| MS-5 ℃_6 h_1 | 42465804 | 42178656 | 0.0243 | 98.28 | 94.81 | 50.4 | Replicate 1 |
| MS-5 ℃_6 h_2 | 44920284 | 44676588 | 0.0232 | 98.74 | 96.04 | 50.21 | Replicate 2 |
| MS-5 ℃_6 h_3 | 46046954 | 45642406 | 0.0202 | 98.42 | 95.29 | 50.24 | Replicate 3 |

## Table S4. The expressed genes and transcripts were analyzed by functional database annotation (NR, Swiss-Prot, Pfam, COG, GO and KEGG).

| Database | Express gene number (percent) | Express transcript number (percent) | All gene number (percent) | All transcript  number (percent) |
| --- | --- | --- | --- | --- |
| GO | 4515 (0.7806) | 4480 (0.7808) | 4530 (0.7527) | 4530 (0.7527) |
| KEGG | 3186 (0.5508) | 3158 (0.5504) | 3191 (0.5302) | 3191 (0.5302) |
| COG | 1156 (0.1999) | 1147 (0.1999) | 1166 (0.1938) | 1166 (0.1938) |
| NR | 5778 (0.999) | 5735 (0.9995) | 5836 (0.9698) | 5836 (0.9698) |
| Swiss-Prot | 4427 (0.7654) | 4393 (0.7656) | 4436 (0.7371) | 4436 (0.7371) |
| Pfam | 4642 (0.8026) | 4609 (0.8032) | 4659 (0.7742) | 4659 (0.7742) |
| Total  annotation | 5778 (0.999) | 5735 (0.9995) | 5836 (0.9698) | 5836 (0.9698) |
| Total | 5784 (1.0) | 5738 (1.0) | 6018 (1.0) | 6018 (1.0) |

## Additional file 1: Table S5. Identified genes for MS612 by comparative transcriptome technology.

## Additional file 2: Table S6. Identification of differentially expressed genes in MS612 at different temperatures and time points.

## Additional file 3: Table S7. Gene Ontology of proteins (GO) annotation of differentially expressed genes in MS612 at different temperatures and time points.

## Additional file 4: Table S8. Kyoto Encyclopedia of Genes and Genomes (KEGG) annotation of differentially expressed genes in MS612 at different temperatures and time points.

## Additional file 5: Table S9. The list of putative candidate genes after cold stress in MS612.
